# Mycobacteriophage D29-mediated lysis improves recovery of mycobacterial genomic DNA from low-biomass samples

**DOI:** 10.64898/2026.08.08.743631

**Published:** 2026-08-08

**Authors:** Joseph Wambugu Gitari, Anastasia Koch, Elizabeth Kigondu, Digby F. Warner, Mandy K. Mason

## Abstract

**Background:**

Detection of rare mycobacterial genotypes, including those associated with antibiotic resistance or population heterogeneity is important for diagnostic, therapeutic and research applications. This depends on efficient recovery of genomic DNA (gDNA) from sampled populations, a challenging requirement in paucibacillary clinical materials. Mycobacteria have uniquely lipid-rich, structurally robust cell envelopes which resists cell lysis by conventional methods. Here, we characterize mycobacteriophage D29-mediated lysis at the single-cell level, evaluating its utility as a biological lysis strategy for mycobacterial DNA isolation, benchmarked against the standard cetyltrimethylammonium bromide (CTAB) extraction method.

**Methods:**

Conditions for mycobacteriophage D29 infection of *Mycobacterium smegmatis* ( *Msm* ) were established, and single-cell phage adsorption and phage-mediated lysis visualized through live-cell time-lapse fluorescence microscopy (FM). A mycobacteriophage D29-based lysis method was applied to both *Msm* and *M. tuberculosis* ( *Mtb* ), and extraction efficiencies compared with the standard CTAB method. Cell lysis efficiency was quantified by colony forming units (CFU), flow cytometry (FC) and FM; DNA yield was determined by quantitative polymerase chain reaction (qPCR) and droplet digital PCR (ddPCR).

**Results:**

Mycobacteriophage D29 adsorption was observed at the poles and septa of individual mycobacterial cells. Phage infection was associated with loss of cytoplasmic green fluorescence protein (GFP) reporter protein, with uptake of a cell death marker propidium iodide (PI). Mycobacteriophage D29 infection resulted in a marked loss of cell viability, with >6log _10_ reduction in CFU, and cell lysis efficiencies calculated as 93.3% (FC) and 96.8% (FM). Molecular quantification (qPCR and ddPCR) indicated that the mycobacteriophage-based lysis achieved between 4- to 7-fold greater gDNA yields in *Msm* and between 3- to 12-fold greater gDNA yields in *Mtb* H37Ra compared with the CTAB method. Notably, gDNA extraction efficiencies in both mycobacterial species exceeded 92% in low-biomass samples containing approximately 100, 175 and 320 bacilli.

**Conclusion:**

These results demonstrate the utility of the mycobacteriophage D29-based method for improved DNA extraction yields from mycobacteria through direct lysis of individual bacilli, with performance suited to low-biomass samples.

**Summary:**

Recovering genomic DNA (gDNA) from low numbers of mycobacteria is a persistent bottleneck for diagnostics and genomic studies, because the lipid-rich mycobacterial envelope resists conventional lysis. Here we show that mycobacteriophage D29 provides an efficient, biologically selective route to mycobacterial DNA. Leveraging single-cell live imaging, we reveal that phage D29 adsorbs preferentially at the poles and septa of individual cells, and that infection is heterogeneous and asynchronous, progressing from envelope permeabilization to loss of viability. Applied as an extraction method and benchmarked against the standard cetyltrimethylammonium bromide (CTAB) protocol, phage D29-mediated lysis recovered 4- to 7-fold more gDNA in *Mycobacterium smegmatis* ( *Msm* ) and 3- to 12-fold more in *Mycobacterium tuberculosis* ( *Mtb* ). Critically, extraction efficiency exceeded 92% in both species in low-biomass samples of approximately 100, 175 and 320 bacilli, where CTAB performed poorly (<20% efficiency). These findings support phage-mediated lysis as a quantitative, near-complete DNA-recovery method that outperforms conventional extraction precisely in the paucibacillary regime of greatest clinical relevance and demonstrate the value of single-cell interrogations in building towards precision tools to engage the mycobacterial cell.

## Full Text Availability

The license terms selected by the author(s) for this preprint version do not permit archiving in PMC. The full text is available from the preprint server.

